# A Synergistic Flame Retardant System Based on Expandable Graphite, Aluminum (Diethyl-)Polyphospinate and Melamine Polyphosphate for Polyamide 6

**DOI:** 10.3390/polym13162712

**Published:** 2021-08-13

**Authors:** Florian Tomiak, Angelina Schoeffel, Klaus Rathberger, Dietmar Drummer

**Affiliations:** 1 Institute of Polymer Technology, Friedrich-Alexander-University Erlangen-Nuremberg, Am Weichselgarten 9, 91058 Erlangen, Germany; dietmar.drummer@fau.de; 2 Bavarian Polymer Institute, Friedrich-Alexander-University Erlangen-Nuremberg, Dr. Mack Strasse 77, 90762 Fuerth, Germany; 3 Georg H. Luh GmbH, Schoene Aussicht 39, 65396 Walluf, Germany; angelina.schoeffel@luh.de (A.S.); klaus.rathberger@luh.de (K.R.)

**Keywords:** nylon 6, PA6, aluminum (diethyl-)polyphosphinate, AlPi, melmanin polyphosphate, MPP, expandable graphite, flame retardant multi-material system, synergistic effect, heating rate

## Abstract

Expandable graphite (EG), aluminum (diethyl)polyphosphinate (AlPi) and melamine polyphophate (MPP) was used as flame retardant multi-material additive in a polyamide 6 (PA6) matrix. Flame inhabitation performances were conducted by cone calorimeter, LOI and UL-94 tests, synergisms identified analyzed by TGA-FTIR and TGA-GC/MS and effects found were comprehensively discussed. SEM images were used for char residue characterization. For PA6 containing 20 wt.% EG and 5 wt.% AlPi/MPP (3:2), a well working synergism in limiting oxygen indices could be identified exhibiting the highest oxygen index (OI) measured: 46%. The study shows that the synergism due to the partial substitution of EG by AlPi/MPP can be attributed to two effects: (1) When PA6/AlPi/MPP mixtures decompose predominantly CO_2_ evaporates in early decomposition stages. CO_2_ evaporations was found to be sensitive to the heating rate applied, whereas specifically high heating rates increased the CO_2_ yield measured. (2) Solid decomposition products of AlPi/MPP act as “glue” between expanded graphite and thus increase the mechanical residue stability.

## 1. Introduction

Polyamide 6 (PA6) is one of the most important representatives of engineering polymers and used in a wide range of technical applications. However, the naturally low flame resistance of most polyamides requires flame-retarding modifications, especially when used in fire-critical applications. For polyamides, a wide range of effective, commercially available flame retardant additive exist, whose effectiveness as an additive alone and in combination with synergists has been studied comprehensively. Particularly successful are solutions based on phosphorus-containing main additives, such as aluminum (diethyl-alkyl). (diethyl) polyphosphinate (AlPi) [1,2], melamine polyphosphate (MPP) [1,3], red phosphorus, ammonium polyphosphate (APP) [4,5] as individual components or combined with other additives, e.g., zinc borate [3,6], kaolin [7], zeolite, etc. in synergistic multi-material systems. Using synergistic material combinations is the most effective way to reduce the overall flame retardant content and therefore a desired possibility to improve material performances [8,9].

Within this paper we discuss an efficient multi-material flame retarding system for polyamide 6 based on expandable graphite (EG) as main ingredient as well as AlPi/MPP mixtures as synergistic component. EGs have been proven to be an excellent flame retardant additive in various polymers as individual component and as multi-material flame retardant system, e.g., PE [10,11,12,13], PP [14,15], PS [16], PVC [17], ABS [18], PA6 [19]. When a critical temperature is reached, blowing agents intercalated between the graphene layers react, forming a voluminous and thermally stable char-residue with a typically worm-like structure. The residue acts as a physical barrier between the yet undecomposed polymer and the gas phase, effectively reducing the exchange rate of heat impact and pyrolysis gas flow. Polymers containing EGs have been proven particularly efficient in fully developed fires by drastically reducing fuel supply due to the thermal-barrier effect, but show less sufficient properties when it comes to flammability and self-extinguishing tests. The worm-like structure is also typically not very stable and, therefore tends to crumble, which might cause fuel leaks and thus reducing the barrier performance. Accordingly, EGs are typically used in combination with synergists, designed to stabilize the residue and lowering flammability limits.

The effect of AlPi and MPP as flame retardant additives in GF and non-GF polyamides has been studied. In PA6 AlPi works through two reaction pathways. A part of the AlPi vaporizes and acts though flame inhabitation and radical elimination. Another part reacts with the polymer to form phosphinic acid, which subsequently leads to phosphoric char formation. AlPi is reported to work for both non and glass-fiber reinforced PA6, but the overall performance is better in non-reinforced systems [1].

MPP performs less effective in PA6 due to the depolymerization mode of decomposition [20] and is, therefore mostly used as synergist in multi-material systems [21]. MPP is reported to be an effective synergist with multiple phosphor-based additives, whereas for glass fiber reinforced PA6/AlPi/MPP combinations show good performances in Cone Calorimeter, limiting oxygen index (LOI) and UL-94 fire tests. When AlPi and MPP are combined, the decomposition process changes significantly. AlPi vaporization is reported to be of less importance for the flame inhibiting reaction, whereas phosphinic acid is released into the gas phase and aluminum phosphate remains in the residue [3].

Within this paper, we present the flame retardant performance and decomposition processes of a multi-material system based on EG, AlPi and MPP in a PA6 matrix. Fire behavior and flame inhibition performance is characterized by cone calorimeter, LOI and UL-94 burning tests. To describe the decomposition pathways TGA-FTIR and TGA-GC/MS measurements are used, interpreted and fire retarding modes of actions discussed. Standard TGA measurements are typically carried out at heating rates of ≤20 K/min. Lower heating rates fundamentally enable better solubility of the gas phase components, but are far from environmental conditions found in real fires. In cone calorimeter tests heating rates of several hundred degrees have been reported [22,23]. For formulations tested within this study we identified a significant dependence of the decomposition behavior on the heating rate applied. Accordingly, TGA-GC/MS analyses were carried out using two heating rates 20 K/min and 100 K/min. Synergistic effects were mostly found in LOI tests for formulations containing EG as main ingredient, as well as AlPi/MPP mixtures at a ratio of 3:2 as synergist.

## 2. Materials and Methods

### 2.1. Materials and Preparation

Materials used in this study was a standard PA6 grade, an expandable graphite type and two conventional phosphorus-based flame retardant additives. The PA6 grade B27E (density 1.13 g/cm^3^; MVR 130 cm^3^/10 min at 275 °C/5 kg) was provided by BASF AG (Ludwigshafen, Germany). A new expandable graphite type GHL PX 95 HT 270 (70% > 50 mesh; pH 5–9) was provided by LUH GmbH (Walluf, Germany). Both phosphorus-based flame retardant additives are halogen free grades MP200 (density 1.85 g/cm^3^; pH 5.5–6.5; particle size d_50_: 10 µm; nitrogen 42.0–44.0%; phosphorus 12.0–14.0%; low water solubility) from BASF AG (Ludwigshafen, Germany) and Exolit OP 1230 (density 1.35 g/cm^3^; d_50_: 20–40 µm; phosphorus 23.3–24.0%, low water solubility) from Clariant AG (Muttenz, Switzerland).

The material components were prepared using a twin-screw extruder (co-rotating) DSE ZSE HP 27 from Leistritz GmbH (Nuremberg, Germany) as well as two gravimetrical feeder units. Temperatures were controlled between die and polymer feeder at 230 °C to 220 °C. For multi-material mixtures containing AlPi, MPP and EG, AlPi/MPP were premixed at a ratio of 3:2 using a universal powder mixer (200 u/min; 15 min). A mixture of 3:2 AlPi/MPP was found to be most efficient in pretrial. The polymer strands were drawn off via a water bath, chipped and dried afterwards. All materials were dried before processing.

To prepare samples for fire testing, plates (115 × 115 × 4 mm^3^) were produced by injection molding using an Arburg Allrounder 370 V injection molding machine from Arburg GmbH & CoKG (Loßburg, Germany). Aggregate temperatures were controlled between 230 °C (die) to 220 °C; injection speed at 60 mm/s; mold temperature at 80 °C. Sample geometries were prepared by sawing and milling. Geometries prepared were as follows: Cone calorimeter samples 100 × 100 × 4 mm³; LOI samples 125 × 10 × 4 mm³; UL-94 125 × 13 × 4 mm³.

#### 2.1.1. Fire Testing

Samples were compounded, injection molded and geometrically modified according to test requirements. Fire testing was conducted using a Cone Calorimeter (ISO 5660-1), a UL-94 (DIN EN 60695-11-10/20) burning chamber and a Limiting Oxygen Index (LOI) (DIN EN ISO 4589-2) device from Netzsch Taurus GmbH (Weimar, Germany). Cone Calorimeter tests provide a comprehensive insight into fire behavior of polymeric materials and offer key figure-based comparability of polymer materials. The most important parameters determined are the heat release rate (HRR), total heat emitted (THE), mass loss rate (MLR) and smoke production rate (SPR). Ratios, average or maximum Values of these parameters, such as the average rate of heat emission (AHRE) or the maximum AHRE (MAHRE), average mass loss rate (AMLR), average specific extinction area (ASEA), total smoke production (TSP) are used for comparative reasons. Key figures assessing fire growth “fire growth rate” (FIGRA), which is defined as maximum HRR(t)/t and “flame spread” fire propagation index (FPI), which is defined as t_ign_/pHRR, where also used within the discussion. For tests conducted within this study 100 × 100 × 4 mm^3^ samples were prepared and tested at a heater power of 50 kW/m^2^. All tests were repeated three times. Evaluation procedures followed the guidelines and recommendations of good scientific practice described in [8,9].

Limiting oxygen index (LOI) tests are commonly used to characterize flammability properties at a given oxygen/nitrogen atmosphere. A 50 W propane flame is applied six times for 5 s each in a candle-like setup, whereas the LOI value represents the highest oxygen content where a visible flame can just be sustained. The LOI value is predominantly used as a comparative value. Specimen geometries were 125 × 10 × 4 mm^3^.

The UL-94 test is used to conduct self-distinguishing properties. Specimens are clamped vertically in a holder and exposed to a 50 W test flame for two times 10 s, each at the lower end. Results are described in three classifications V-0, V-1, V-2, whereas V-0 represents an instant self-extinguishing without or with non-burn-dripping behavior. V-1 classifications indicate slightly longer self-extinguishing times, whereas V-2 classifications show slow self-extinguishing behavior that might be accompanied by burn dripping. Specimen geometries used within this paper are 125 × 13 × 4 mm^3^. All tests were conducted in accordance with standards. All samples were dried 7 days in vacuum at 70 °C before testing.

#### 2.1.2. Decomposition Analytics

Decomposition behavior was studied by using coupled thermogravimetrical analysis (TGA)-Fourier-transformation-infrared-spectroscopy (FTIR) as well as TGA—gas chromatography and mass spectrometer (GC/MS) under nitrogen atmosphere. A TGA device STA F3 449 Jupiter from Netzsch (Selb, Germany) equipped with a high-speed oven as well as a TG-DSC sample carrier was used. TGA measurements were conducted using aluminum oxide tilts. Samples were heated from 50 to 800 °C. The heating rate applied was 20 K/min; the gas flow applied was 70 mL/min. Sample weights were kept constant at 10 ± 1 mg (20 K/min). The onset temperature is defined as 99% residual mass. All tests were conducted three times whereas averaged curves are presented. The FTIR unit Tensor 2 from Bruker Corp. (Billerica, MA, USA) was coupled by a 230 °C controlled transfer line. FTIR gas-cell temperatures were controlled at 200 °C; 32 scans were averaged. A 30 s measurement delay occurred between TGA and FTIR results, which corresponds to 10 °C.

Coupled TGA-GC/MS was used to quantify molecular gas phase fractions. Heating rates applied were 20 K/min and 100 K/min; gas flows applied were 70 mL/min and 120 mL/min. Temperature calibration was carried out professionally using melting temperatures of calibration metals. Sample weights were kept constant at 10 ± 1 mg (20 K/min) and 5 ± 1 mg (100 K/min). No temperature shifts due to mass inertia were detected. The transfer line was controlled at 280 °C. A valve box equipped with two sampling loops from Netzsch GmbH (Selb, Germany) was used for reciprocal column loading every 10 s during decomposition. The valve box was temperature controlled at 300 °C. The GC (7890B) and MS (5977B/MSD) device were from Agilent Technologies Inc. (Santa Clara, CA, USA). The purge gas was helium; the column length was 30 m. For sample collection, a cryo-trap was operated at −160 °C; the oven program subsequent to sample collection heated the cryo-trap in 10 K/min increments; the GC oven was heated from 40 °C to 180 °C with a ramp of 2 K/min, to ensure proper peak-separation. Sampling programs used represent a cumulative preceding, always starting before the decomposition onset and ended at the relative mass loss specified. All tests were repeated three times.

#### 2.1.3. Microscopy

To identify the overall particle shape scanning electron microscope (SEM) studies were performed. The powder samples are spattered with a platinum-palladium mixture. A SEM Ultra Plus system from Zeiss (Oberkochen, Germany) was used for the analysis.

## 3. Results

### 3.1. Thermal Decomposition

Regardless of the flame retardant additives added to PA6, lower onset temperatures and different gravimetrical decomposition characteristics were identified. Higher heating rates naturally shift the decomposition process towards higher temperatures, whereby the general decomposition characteristic does not change for all formulations used within this study. For the following description values conducted from TGA heating rates of 20 K/min are named first, of 100 K/min are named in brackets. The temperature delta is marked as ∆ and also given in brackets. Please note the standard deviations are given in Table 1.

The decomposition behavior of PA6 was characterized as one single step, marking an onset temperature at 393 °C (433 °C; ∆ 40 °C) and a maximum mass loss rate at 477 °C (510 °C; ∆ 33 °C). As expected, no charring occurred giving a residue of 0.8% (0.9%; ∆ 0.1%) at 800 °C. Literature data provide similar results [24,25]. When expandable graphite (EG) is added, the decomposition onset temperature decreases significantly to 313 °C (338 °C), but decomposition subsequently continues at a lower mass loss rate. A blowing agent intercalated in between graphene layers causes the early onset temperature. No TGA curve indication for potential interactions between polymer and EG could be identified, thus the temperature conducted at a maximum mass loss rate of 466 °C (504 °C; ∆ 38 °C) differs only slightly from net PA6. Char residue at 800 °C mainly corresponds to graphite with 25.5% (25.0%; ∆ 0.5%).

**Figure 1:**
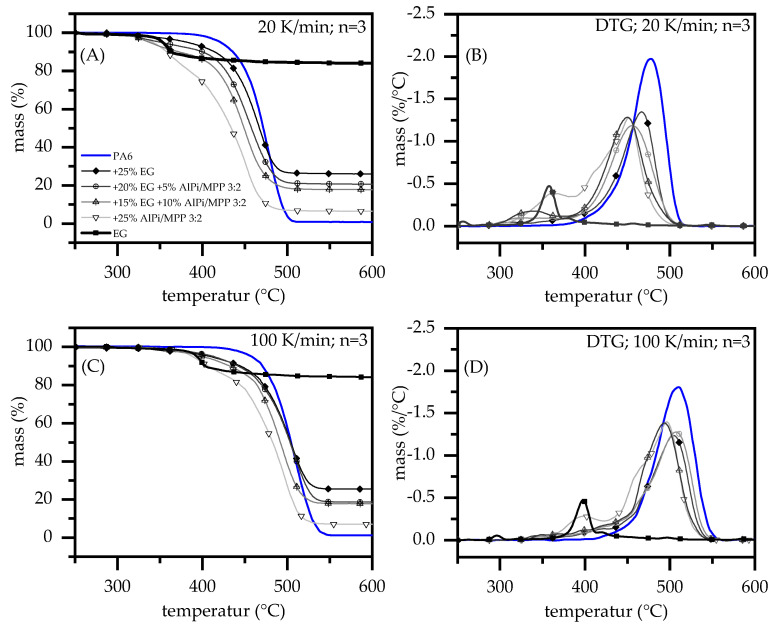
TGA Measurements of PA6 containing 25 wt.% expandable graphite, aluminium (diethyl-)polyphosphinat, melamin polyphosphat as well as a 3:2 mixing ration AlPi/MPP; Comparison to a multi-material system including EG, AlPi and MPP under nitrogen atmosphere. TGA heating rate 20 K/min (**A**,**B**) and 100 K/min (**C**,**D**).

AlPi causes the smallest temperature shifts (Figure 1). When the onset temperature of 383 °C (425 °C; ∆ 42 °C) is reached, mass decreases rapidly at rates comparable to net PA6. However, mass loss peaks 468 °C (486 °C; ∆18 °C) tend to appear at relatively lower temperatures for a 100 K/min, indicating an acceleration of decomposition kinetics. Little charring occurs giving also a slightly differing residual mass of 3.8% (5.0% ± 1.2%) at 800 °C. The decomposition process for PA6 containing MPP (Table 1) starts significantly earlier at onset temperature of 334 °C (372 °C; ∆ 38 °C) and decomposes in two gravimetrical steps at 391 °C and 428 °C (425 °C; ∆ 34 °C and 462 °C; ∆ 34 °C). When the first mass loss peak is reached 38% (38%; ∆ 0%), at the second mass loss peak 75% (75%; ∆ 0%) of the initial mass is pyrolyzed. However, strong charring occurs leaving a remaining char residue of 11.7% (11.5%; ∆ 0.2%). No heating rate dependency of the decomposition process could be identified. For reasons of clarity, TGA curves for PA6/MPP and PA6/AlPi have not been plotted. Characteristic key figures are summarized in Table 1.

By combining AlPi and MPP in PA6 matrix using a ratio of 3:2, different curve characteristics occur. The onset temperature of 307 °C (364 °C; ∆ 57 °C) is certainly lower than individual AlPi and MPP formulations. Two clearly distinguishable decomposition stages occur forming a peak mass loss rate at 361 °C and 451 °C (399 °C; ∆ 38 °C and 497 °C; ∆ 46 °C), marking chemical interactions between both additives. Another, non-clearly distinguishable step occurs in DTG curves forming an intermediate shoulder between the first and second step. At 800 °C a char residue of 6.3 % (6.7%; ∆ 0.4%) was identified, which is only slightly higher than an expected value of 7.0% (7.6%; ∆ 0.6%).

As a combination of all three additives, two formulations were combined, each with a main content of 15 wt.% and 20 wt.% EG, as well as a 3:2 mixture of AlPi/MPP at 10 wt.% and 5 wt %, respectively. As follows, the formulations are referred to as Nr. 7 and Nr.6. Both material formulations essentially follow similar gravimetric decomposition characteristics as the formulation with pure EG indicating a single step process. The onset temperature is identical to the PA6/EG formulation with (Nr.6) 309 °C and (Nr.7) 311 °C (354 °C; ∆ 45 °C and 350 °C; ∆ 41 °C), whereas the formulation Nr. 7 decomposes more rapidly. Accordingly, the mass loss peak is reached earlier at (Nr.7) 450 °C (495 °C; ∆ 45 °C) compared to Nr.6 at 457 °C (509 °C; ∆ 52 °C). Thus, a slight increase in char temperature stability can be identified. The remaining mass at 800 °C for Nr.6 with 20.3% (18.2%; ∆ 2.1%) and Nr.7 with 17.4% (17.4%; ∆ 0%) correspond to calculated values 21.7% and 17.8%.

### 3.2. Evolved Gas Analysis—TGA-FTIR and TGA-GC/MS

**Figure 2:**
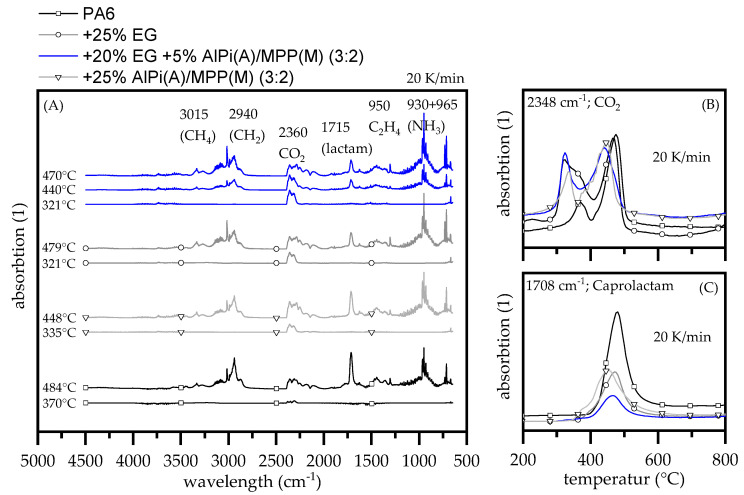
Comparison of pyrolysis gas evolved (FTIR) using TGA heating rates of 20 K/min conducted under nitrogen atmosphere (**A**). Traces of (**B**) CO_2_ and (**C**) Caprolactam were extracted and plotted for evaluation. Samples were taken from injection molded plates.

Decomposition pathways were studied by TGA-FTIR measurements at a heating rate of 20 K/min (see Figure 2). Since we found major differences in the quantitative development of individual gas phase components observable over the decomposition progress, additional TGA-GC/MS measurements were used to quantify changes in certain temperature ranges. We analyzed quantitative changes for heating rates of 20 K/min and 100 K/min by systematic exhaust gas sampling using a cryo-trap as well as a specific GC-oven program for sufficient peak detection.

Net PA6 mostly decomposes to its monomer caprolactam and subsequent decomposition products, which is not only fast at high temperatures, but also volatile [26]. FTIR gas phase analytics revealed two decomposition peaks at 370 °C and 484 °C, whereas the first one indicates only CO_2_ release. CO_2_ production in PA6 decomposition is generally assumed to origin from hydrolytic scission of the C(O)-NH bond and the subsequent decomposition of carbon acid to –CO–, CO_2_ and water [24,27]. Since the first CO_2_ release-peak does not refer to any mass loss step seen in TGA measurements, we attributed this to some early decomposition effects, whereas no specific origin could be assigned. The second step corresponds well to the main gravimetrical step seen in TGA measurements at 477 ± 2 °C, considering a slight time gap between pyrolysis and FTIR gas chamber. Gas products reported in the literature for PA6 decomposition in FTIR measurements [28,29,30] were also found: caprolactam (1715 cm^−1^ and fingerprint pattern 1305, 1352, 1361 as well as 2865 cm^−1^), ammonia (930, 965 cm^−1^), NH groups (3334 cm^−1^), CH_2_ groups (2940, 2865, 1440 cm^−1^), ethene (950 cm^−1^), methane (3015 cm^−1^ fingerprint pattern 1200–1400 cm^−1^), CO_2_ (2360, 671 cm^−1^), CO (2114, 2174 cm^−1^) and water (3853, 3400–4000 cm^−1^).

Modes of fire retarding actions of AlPi and/or MPP have been studied as flame retardant additive within glass fiber reinforced PA6 [1] and PA6.6 [3]. AlPi is reported to partially vaporize and react with the polyamide, forming phosphoric acid and phosphoric char, thus contributing to the flame inhabitation effect both in gas and condensed phase. PA6 containing MPP shows a different decomposition behavior. As reported in the literature, MPP accelerates the decomposition process in PA6 and forms a phosphinic char with no gas phase action.

When AlPi and MPP is combined, the decomposition process changes. AlPi vaporization is reported to be of less importance for the flame inhibiting effect. Some phosphinic acid is released into the gas phase and aluminum phosphate remains in the residue [3]. Similar results were found within this study. FTIR gas phase analytics of PA6 containing AlPi/MPP 3:2 showed two major decomposition steps: (1) at a peak temperature of 335 °C only CO_2_ release was detected, which has also been discovered earlier for net PA6. (2) at a peak temperature of 448 °C, which represents PA6 decomposition. Compared to net PA6 no major gas phase changes could be detected for the second decomposition step, indicating no specific flame retarding gas phase mode of action given by the additive.

EG filled PA6 also showed two decomposition steps. The first one also reveals a CO_2_ peak (335 °C), but appears even earlier than identified from PA6/AlPi/MPP formulations. Expandable graphites slowly starts to expand from temperatures > 270 °C, thus create larger surfaces shortening heat to reaction times. On the other hand, the main decomposition step of PA6 appears at higher temperatures 479 °C, which is very close to that of net PA6 at 484 °C. Since TGA curves fit well to the individual materials, we assume independent decomposition processes marking nonchemical interactions.

Formulations containing PA6 + 25 wt.% EG as well as a 5 wt.% AlPi/MPP (3:2) mixture basically combine all individual reaction profiles discussed: (1) CO_2_ release at 321 °C equals the profile given by PA6/EG formulations; (2) partly decomposition of PA6/AlPi/MPP at 440 °C; (3) PA6 decomposition step at 470 °C. No major gas phase changes were identified.

Hardly any deviations in the qualitative gas phase composition were found, though deviating absorption levels indicate differences in the quantitative appearance. Most obvious changes can be identified in caprolactam (1708 cm^−1^), CO_2_ (2360 cm^−1^), ethane (950 cm^−1^) and methane (3015 cm^−1^) traces. Specifically, caprolactam and CO_2_ have been identified to be of particular interest for the resulting flame retarding performance and is therefore discussed in more detail hereinafter. As TGA-FTIR studies have already shown, the decomposition process does not change for PA6+EG+AlPi/MPP formulations compared to the single ingredient formulations. However, the functionality of multi-material flame retardant systems does not depend exclusively on the type of chemical interactions. Particularly when several active ingredients are expected to act synergistically, the timing and intensity of certain reactions is essential. As we found not only timing differences in CO_2_ and caprolactam evaporation, but also quantity changes when different heating rates were applied, TGA-GC/MS was performed using TGA heating rates of 20 K/min and 100 K/min. Molecular proportions of major gas phase proportions are listed in Table 2.

**Figure 3:**
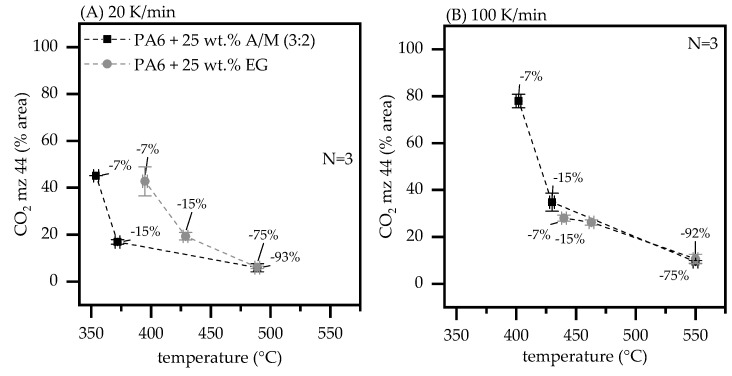
Proportional CO_2_ evaporation (count %) cumulatively measured at TGA mass loss stages of −7%, −15% and at complete combustion −75%/−93%. Sampling was conducted at TGA heating rates of (**A**) 20 K/min and (**B**) 100 K/min under nitrogen atmosphere.

In early decomposition stages PA6/AlPi/MPP and PA6/EG formulations predominantly evaporate CO_2_ and caprolactam as pyrolysis product, whereas for the latter also sulfur dioxide (SO_2_) traces could be detected (Table 2, Figure 3). This fits well to findings in other studies [4,31].

When a heating rate of 20 K/min was applied, both formulations tend to evaporate identical gas phase proportions of CO_2_, measured at TGA mass loss stages of −7%, −15% and total combustion −75%/−93%. However, for PA6 containing 25 wt.% AlPi/MPP (3:2) CO_2_ evaporates both at significantly lower temperatures and at a narrower temperature range than the corresponding PA6/EG formulation (Figure 3A). A narrower temperature range would amplify potential dilution effects caused by CO_2_ evaporation, but limit them to a shorter period of time. With increasing temperature, the CO_2_ yield drops, whereas other molecular gas fractions increase. The proportion of caprolactam evaporation first seems to increase and then to decrease again. This observation fits well to findings in another study [31].

When a heating rate of 100 K/min was applied, the CO_2_ gas phase proportion specifically in early decomposition stages seems to develop differently for both formulations.

For TGA mass losses of −7% PA6/AlPi/MPP formulations yield a CO_2_ proportion of 78%, whereas PA6/EG formulations only yield 28%. For the latter, an increasing dominance of other hydrocarbonates (besides caprolactam) in the gas phase can also be identified. Since the total concentration remains identical at complete combustion within standard deviation and no chemical effects indicate a change in reaction pathways, we attribute this effect to a quicker CO_2_ formation as well as faster physical transportation caused by lower density. High CO_2_ yields are of particular importance for dilution flame inhibiting effects. The “fuel inertization point” of hydrocarbonates caused by delusion can only be achieved at very high volume fractions of inert gases. A comprehensive study has numbered the volume fraction CO_2_ for a number of hydrocarbonates to well over 80 vol.%. [32]. Please note this number can only be taken for orientation purposes. Due to the low density in relation to caprolactam, CO_2_ volumetrically dominates the gas phase in early decomposition stages. However, with ongoing decomposition and other hydrocarbons entering the gas phase, the dilution effect gradually disappears.

### 3.3. Burning Behavior—Cone Calorimeter, LOI and UL-94

The net heat of combustion for PA6 measured in cone calorimeter tests was 26.0 ± 1.0 kJ/g, which corresponds well to literature values for the net heat of complete combustion of 28.8 ± 1.1 kJ/g [33]. The delta can be attributed to some melt dripping.

**Figure 4:**
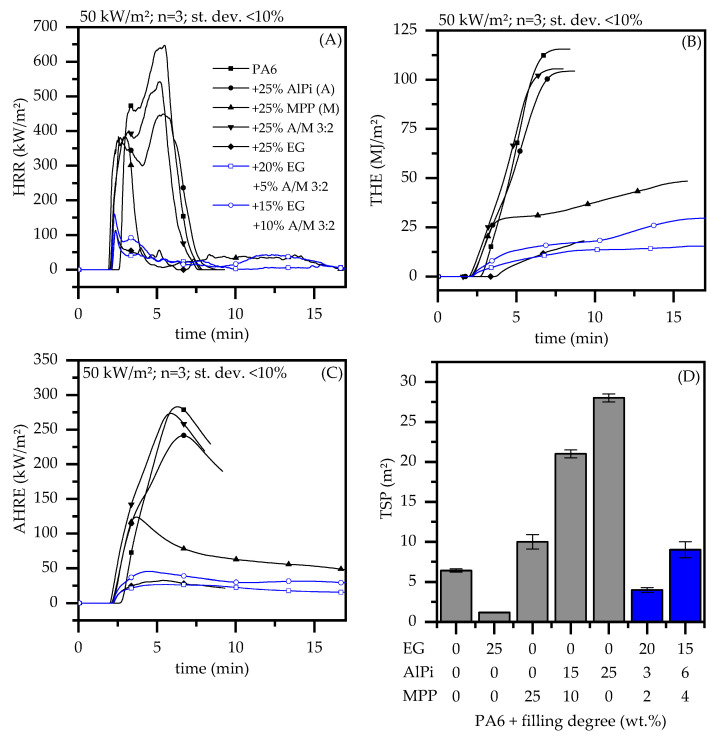
(**A**) Heat release rate (HRR); (**B**) Total heat release rate; (**C**) average rate of heat released (AHRE) and (**D**) Total smoke production (TSP) for various PA6 recipes; cone heater 50 kW/m^2^; dry samples.

Polymers modified with expandable graphites usually show strong flame retardant properties in fire tests particularly sensitive to residue barrier formation, but not necessarily good performance in flammability tests (Figure 4, Table 3). Compared to net PA6, the pHRR is reduced from 648 kW/m^2^ to 120 kW/m^2^ for 25 wt.% loading levels of EG as well as the corresponding MAHRE values from 289 kW/m^2^ to 33 kW/m^2^. This reduction is significant, especially when compared to non-charring PA6 formulations containing 25 wt.% AlPi or 3:2 AlPi/MPP showing pHRR of 464 kW/m^2^ and 563 kW/m^2^ as well as MAHRE values of 242 kW/m^2^ and 274 kW/m^2^. Considering THE, the reduction of PA6 containing 25 wt.% EG versus net PA6 drops from 115 MJ/m^2^ to 12 MJ/m^2^. This can be explained by a very low burning rate. In fact, the burning rate was in parts too low to be detected by the O_2_ analytics. A low heat generation is also reflected in comparative key figures FIGRA and FPI, which are used to assess fire growth and flame spread. Compared to all tested formulations, EG-filled PA6 exhibiting a FIGRA of 0.8 kW/(m^2^*s) and a FPI of 1.1 (s*m^2^)/kW represent the lowest tendencies of contributing to emerging fires. Additionally, almost no visible smoke generation could be detected represented by a TSP of 1.2 m^2^.

Considering PA6 modified by 25 wt.% AlPi or AlPi/MPP 3:2 as single ingredient, the reduction in THE is less sufficient reaching values of 104 MJ/m^2^ and 105 MJ/m^2^. Due to no residue formation, both additives tend to decompose quickly, whereas formulations containing AlPi/MPP 3:2 even accelerate the decomposition process.

For PA6/MPP formulations a special heat development characteristic can be identified. Although MPP forms a protective residue, chemical reaction of residue formation occurs in advanced burning-stages. Until an effective barrier is formed, the fire development proceeds unstopped, marking a temporary pHRR of 402 kW/m^2^. From the moment a critical residue is formed, a significant decrease in heat development can be observed.

Although AlPi/MPP ratios do not show sufficient flame retardancy effects as individual ingredient in PA6, we found a well working synergism when combined with PA6/EG. For this purpose, a formulation with PA6 and 25 wt.% EG was gradually substituted with 5 wt.% and 10 wt.% of an AlPi/MPP 3:2 mixture. Preliminary studies showed that most chemical interactions can be observed for 3:2 ratios AlPi/MPP, thus no further ratio-variations were conducted. The investigations revealed a clear synergism for LOI tests, while a V-0 classification remained and a slight decline in most cone calorimeter key figures could be observed. Variations in global loading levels were not conducted.

**Figure 5:**
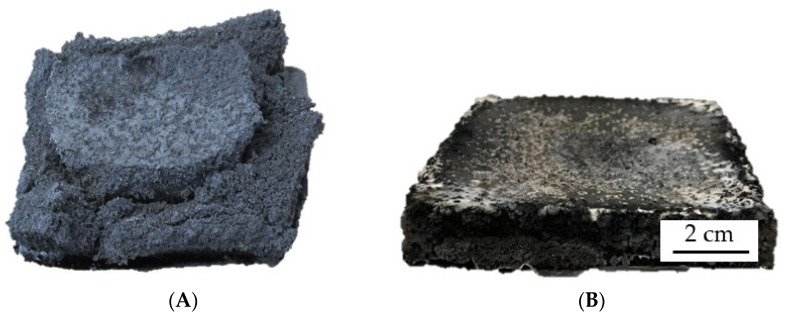
Residues after cone calorimeter testing (**A**) PA6 + 25 wt.%; (**B**) PA6 + 15 wt.% EG + 10% AlPi/MPP 3:2.

Despite PA6/EG/AlPi/MPP compared to PA6/EG formulations do slightly decrease most performance figures in cone calorimeter tests, the decrease in performance is relatively low when compared to PA6 containing 15 wt.% EG (pHRR: 265 kW/m^2^; THE 84 MJ/m^2^; MAHRE 130 kW/m^2^). As already discussed, AlPi/MPP as a single ingredient hardly contributes to a flame-resisting characteristic in cone calorimeter tests. Although a partial substitution of EG in PA6/EG in favor of AlPi/MPP equivalently reduces the protective barrier effect, a beneficial contribution regarding the mechanical residue stability can be identified (Figure 5). This can be particularly advantageous in non-horizontal test setups.

**Figure 6:**
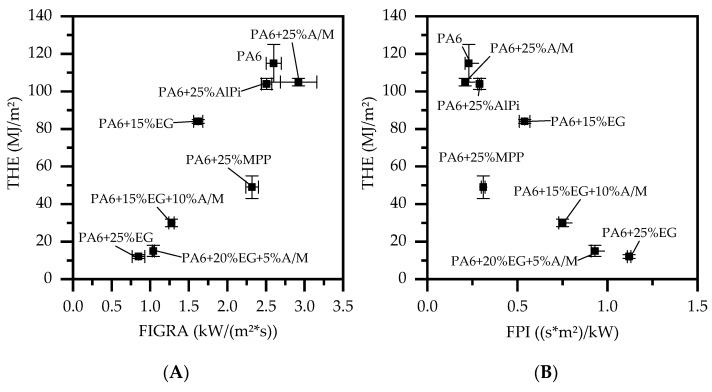
(**A**) FIGRA (ratio maximum HRR(t)/t) plot versus THE and (**B**) FPI (T_ign_/pHRR) plot versus THE.

The fire growth rate (FIGRA) and fire propagation index (FPI) indicators compress complex burning characteristics measured in cone calorimeter tests into one single key figure and are used for comparative reasons only. Higher FPI ratios, as well as lower FIGRA ratios, indicate improved fire protection properties (see Figure 6). For PA6 formulations containing 20/15 wt.% EG and 5/10 wt.% AlPi/MPP 3:2 FIGRA/FPI deteriorate slightly from 0.9 kW/(m^2^*s)/1.1 (s*m^2^)/kW for PA6/EG to 1.0 kW/(m^2^*s)/0.9 (s*m^2^)/kW and 1.3 kW/(m^2^*s)/0.8 (s*m^2^)/kW, respectively, which may be acceptable in view of the improvements in residue stability and LOI. Also, pHRR and THE were 144 kW/m^2^ and 15 MJ/m^2^ and 169 kW/m^2^ and 30 MJ/m^2^. The MAHRE value for PA6 containing 20 wt.% EG and 5 wt.% AlPi/MPP 3:2 measured was 28 kW/m^2^ and therefore even somewhat superior to the value measured for PA6/EG formulations: 33 kW/m^2^.

**Figure 7:**
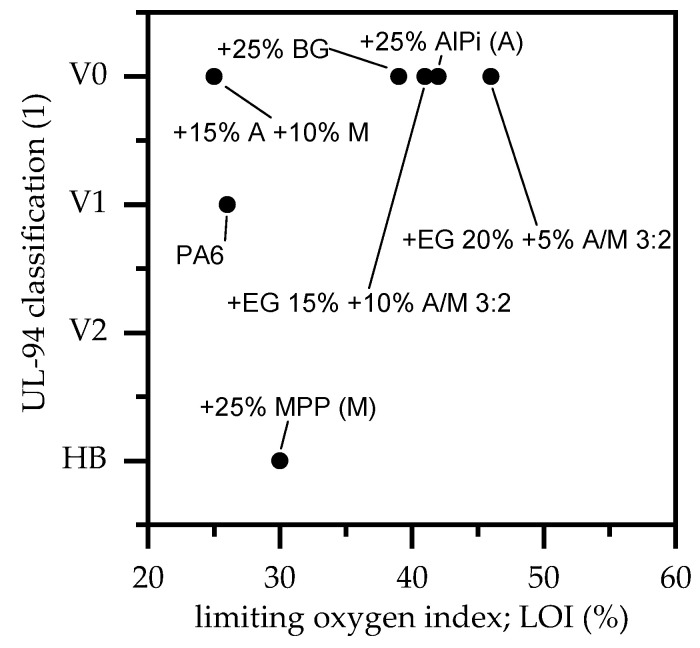
LOI and UL-94 classifications; sample thickness: 4 mm.

For LOI and UL-94 fire testing, specimens with a thickness of 4 mm were used. In accordance with the literature, net PA6 achieved a V-2 classification as well as a LOI of 26% (Figure 7, Table 4). The relatively high classifications can be attributed to a low melt viscosity, which leads to burning dripping in UL-94 tests and a vertical melt flow in LOI testing and thus defueling the flame. Amongst all tested single additive formulations, PA6 containing AlPi provided outstanding performance values with an LOI of 42% and V-0 classification. No dripping was observed in both tests. PA6/MPP and PA6/AlPi/MPP, both at a filling level of 25 wt.%, perform poorly in LOI and UL-94 testing. Despite PA6/MPP a relatively high LOI, the UL-94 cannot be passed. Almost no residue forms during the test, leaving the specimen unprotected. On the contrary, PA6/AlPi/MPP formulations do exhibit a significantly lower LOI at 25%, but do pass the UL-94 test achieving a V-0 classification. However, high t1 + t2 burning times suggest lower UL-94 classifications for thinner samples. PA6 containing 25 wt.% EG exceeds an LOI of 39% as well as a V-0 classification. Though, while AlPi content can be easily reduced and still achieves a V-0 classification, lower loading levels of EG also result in lower UL-94 classifications. EG exclusively acts as physical barrier and thus can only affect the pyrolysis flow provided. Flammability limits of decomposition gases are not affected. Accordingly, to pass the UL-94 test, a minimum residue is required to sufficiently suppress pyrolysis gas flows and thus exhibit a self-extinguishing characteristic.

Considering PA6/EG/AlPi/MPP formulations a synergistic effect can be identified. As already discussed, AlPi/MPP mixtures increase the CO_2_ yield and thus act through dilution. Although this effect is not particularly effective as single ingredient in PA6, we exhibited a complementary effect when combined with EG act. Thus, a LOI of 46% and 41% could be achieved for formulations containing 20/15 wt.% EG and 5/10 wt.% AlPi/MPP 3:2, respectively. Self-extinguishing in UL-94 tests occurs almost immediately. No ignition and afterglow could be observed for both mixtures.

### 3.4. Char Residue Analysis

**Figure 8:**
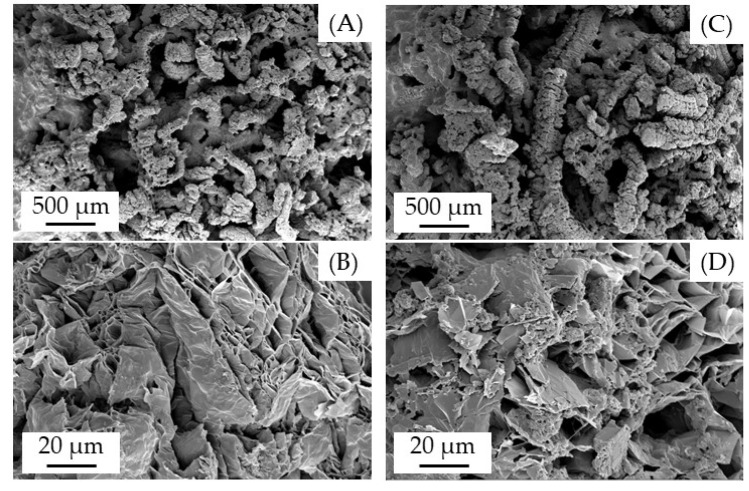
SEM image of UL-94 residues. (**A**,**B**) PA6 + 25 wt.% EG; (**C**,**D**) PA7 + 20 wt% EG + 5 wt.% AlPi/MPP 3:2.

To study the char residue built, SEM images were taken from the pyrolysis zone of UL-94 samples and illustrated in Figure 8. As previously observed in fire tests, formulations containing PA6/EG/AlPi/MPP (Figure 8C,D) versus PA6/EG (Figure 8A,B) produce mechanically more stable char formations in all fire tests conducted. SEM images suggest an additional residue formation through AlPi/MPP interactions acting as “glue” in between graphite particles. The additional residue formation appears as buildup attached to expanded graphite, which optically seem to thicken and appear as bridges.

## 4. Discussion

Fire testing exhibit complementing effects in PA6/EG/AlPi/MPP formulations, which can act synergistically in specific testing scenarios. While PA6/EG formulations perform substantially well in cone calorimeter studies, which simulate a fully developed fire, the same formulation performs less effective in flammability tests LOI and UL-94. EG works as thermal barrier, physically increasing the temperature gradient between flame and polymer. Especially in early decomposition stages, this barrier is not sufficiently developed and thus only little protection is given. Due to no chemical reactions lowering the pyrolysis flammability of the polymer, only high loading levels, which also includes fuel substitution, can offer sufficient protection in flammability tests. AlPi/MPP mixtures have been shown to work well when combined with other flame retardants or glass fibers in PA6, whereas mostly residual effects have been reported in the literature. Similar effects could be found for formulations containing EG in this study. Within all fire tests conducted the residue stability increased significantly (Figure 6), showing a more dense and less crumbly characteristic than PA6 containing solemnly EG. Additionally, we observe a different CO_2_ formation characteristic, effecting time and intensity of evolution, but not the global CO_2_ yield released. As TGA measurements revealed, PA6/AlPi/MPP formulations decompose both at lower temperatures and at a higher decomposition speed. The expansion process of the expandable graphite, which starts at about 300 °C, is thus complementary to the CO_2_ gas evolution through the decomposition process of PA6/AlPI/MPP formulations. Within this temperature range, the physical barrier effect of the expanded graphites is not yet sufficiently developed. Early pyrolysis products of PA6/EG formulations are identical to pure PA6, so that also identical flammability limits can be assumed. We conclude that the complementary CO_2_ yield benefits the barrier-transition stage in PA6/EG/AlPI/MPP formulations by significantly lowering the volume fraction of combustible gas products. As the EG layer expands, dilution effects become less relevant and the thermal barrier dominates the flame inhabitation effect. Higher CO_2_ yields, as discovered in TGA-GC/MS measurements at heating rates of 100 K/min, additionally benefit a short term but more intense dilution effect. However, it must be mentioned, that an early release of CO_2_ in PA6/EG/AlPi/MPP formulations only seems to suppress ignition when the ignition source itself is appropriately low (as in LOI and UL-94). No changes in certain cone calorimeter key figures as the “time to ignition” (t_ign_) or total heat emerged (THE) could be identified.

## 5. Conclusions

Expandable graphite has been shown to be particularly advantageous when used as flame retardant additive for PA6 in cone calorimeter tests. Residues formed by expandable graphite do not have to form chemically, are thermally stable and expand by several hundred times to form an efficient heat barrier between polymer and heat source. Thus flames are only fueled by very low pyrolysis flows, significantly reducing the heat evolved over time. However, due to no chemical effects reducing the lower flammability limit of pyrolysis gases, expandable graphite modified PA6 does not achieve sufficient performances in flammability tests LOI and UL-94. A synergistic multi-material mixture achieving good flame retarding performances was found for PA6/EG/AlPi/MPP formulations. The main assumptions we concluded from this study are summarized as follows:

- PA6 + 20 wt.% EG/5 wt.% AlPi/MPP (3:2) formulations were found to improve the LOI to 46%, whereas only minor performance compromises in cone calorimeter tests must be accepted.
- Two effects have been concluded to work synergistically: (1) A complementary CO_2_ evaporation given by PA6/AlPi/MPP decomposition works efficiently by gas phase dilution, while expandable graphite is still in early expansion stages giving non or little fire barrier. (2) Solid PA6/AlPi/MPP decomposition products also work as “glue” in between expanded graphite, significantly stabilizing the residual char.

## Figures and Tables

**Table 1:** TGA measurement-summary.

			20 K/min			100 K/min		
No.	PA6 wt.%	EG/AlPi/MPP wt.%	t_onset_ (99%) °C	DTG-Peak °C/min	Residue %	t_onset_ (99%) °C	DTG-Peak °C/min	Residue %
1	100	0/0/0	393 ± 2	477 ± 2	0.8 ± 0.1	433 ± 0	510 ± 0	0.9 ± 0.4
	0	100/0/0	307 ± 2	357 ± 2	84.0 ± 0.5	348 ± 2	398 ± 2	84.0 ± 0.5
2	75	25/0/0	313 ± 5	466 ± 5	25.5 ± 0.6	338 ± 2	503 ± 2	25.0 ± 0.5
3	75	0/25/0	383 ± 2	466 ± 2	3.8 ± 0.1	425 ± 0	486 ± 0	5.0 ± 0.0
4	75	0/0/25	334 ± 5	391; 427 ± 5	11.7 ± 0.1	372 ± 0	424; 465 ± 0	11.5 ± 0.2
5	75	0/15/10	307 ± 5	362; 451 ± 5	6.3 ± 0.2	364 ± 7	400; 497 ± 7	6.7 ± 0.1
6	75	20/3/2	309 ± 2	456 ± 2	20.3 ± 0.6	354 ± 4	507 ± 4	18.2 ± 1.7
7	75	15/6/4	311 ± 1	339; 450 ± 1	17.4 ± 0.5	350 ± 0	-; 493 ± 0	17.4 ± 1.7

**Table 2:** TGA-GC/MS—summary of all molecules >5 peak area % (count %).

No.	MZ		20 K/min; Peak Area %			100 K/min; Peak Area %		
**PA6** **+25% EG**		**TGA:**	∆-7% 395 °C	∆-15% 429 °C	∆-71% 489 °C	∆-7% 440 °C	∆-15% 463 °C	∆-71% 550 °C
1	44	CO_2_	52 ± 4%	21 ± 2%	9 ± 3%	28 ± 1%	26 ± 1%	11 ± 2%
2	53	C_3_H_3_N						6 ± 1%
3	54	C_4_H_6_				9 ± 0%	8 ± 0%	6 ± 0%
4	64	SO_2_	11 ± 0%	3 ± 1%	7 ± 1%	3 ± 0%	4 ± 0%	2 ± 0%
5	78	C_6_H_6_				4 ± 0%	5 ± 0%	15 ± 1%
6	113	C_6_H_11_NO	23 ± 2%	66 ± 2%	38 ± 2%	28 ± 1%	30 ± 0%	13 ± 1%
**PA6** **+25% A/M**		**TGA:**	∆-7% 354 °C	∆-15% 372 °C	∆-92% 489 °C	∆-7% 402 °C	∆-15% 430 °C	∆-92% 550 °C
1	41	C_3_H_6_			6 ± 0%		4 ± 0%	4 ± 1%
2	44	CO_2_	50 ± 1%	19 ± 2%	6 ± 1%	78 ± 3%	27 ± 3%	10 ± 1%
3	54	C_4_H_6_			5 ± 1%		10 ± 2%	7 ± 1%
4	56	C_4_H_8_			3 ± 0%		10 ± 2%	
5	78	C_6_H_6_			4 ± 1%			12 ± 1%
6	113	C_6_H_11_NO	33 ± 1%	54 ± 4%	44 ± 3%	13 ± 2%	21 ± 4%	14 ± 1%

**Table 3:** Summary—fire testing results Cone calorimeter; 50 kW/m^2^.

Nr.	PA6 wt.%	EG/AlPi/MPP wt.%	Mass g	t_ign_ s	pHRR kW/m^2^	THE MJ/m^2^	MAHRE kW/m^2^	AMLR g/min	TSP m^2^	FIGRA † kW/(m^2^*s)	FPI †† s*m^2^/kW
1	100	0/0/0	44 ± 1	162 ± 2	648 ± 42	115 ± 10	289 ± 20	4.9 ± 0.3	6.4 ± 0.2	2.6 ± 0.10	0.2 ± 0.02
	85	15/0/0	48 ± 1	143 ± 2	265 ± 10	84 ± 1	130 ± 3	2.7 ± 0.1	5.1 ± 0.1	1.6 ± 0.06	0.5 ± 0.03
2	75	25/0/0	48 ± 1	134 ± 2	120 ± 12	12 ± 1	33 ± 3	1.4 ± 0.1	1.2 ± 0.0	0.9 ± 0.07	1.1 ± 0.01
3	75	0/25/0	45 ± 1	148 ± 2	464 ± 26	104 ± 3	242 ± 11	4.9 ± 0.3	28.0 ± 0.5	2.5 ± 0.07	0.3 ± 0.01
4	75	0/0/25	47 ± 1	126 ± 1	402 ± 15	49 ± 6	124 ± 8	2.2 ± 0.1	10.0 ± 0.9	2.3 ± 0.08	0.3 ± 0.01
5	75	0/15/10	46 ± 1	121 ± 1	563 ± 1	105 ± 2	274 ± 3	5.3 ± 0.0	21.0 ± 0.5	2.9 ± 0.20	0.2 ± 0.00
6	75	20/3/2	48 ± 1	134 ± 3	144 ± 4	15 ± 3	28 ± 3	1.6 ± 0.1	4.0 ± 0.3	1.0 ± 0.02	0.9 ± 0.02
7	75	15/6/4	48 ± 1	127 ± 3	169 ± 10	30 ± 2	47 ± 2	1.9 ± 0.1	9.0 ± 1.0	1.3 ± 0.04	0.8 ± 0.02

† fire growth rate (FIGRA): ratio maximum HRR(t)/t. †† fire propagation index (FPI): T_ign_/pHRR.

**Table 4:** Summary–LOI and UL-94.

Nr.	PA6 wt.%	EG/AlPi/MPP wt.%	LOI %	UL-94
1	100	0/0/0	26	V-2
2	75	25/0/0	39	V-0
3	75	0/25/0	42	V-0
4	75	0/0/25	30	HB
5	75	0/15/10	25	V-0
6	75	20/3/2	46	V-0
7	75	15/6/4	41	V-0
